# Supplementary material for: Complexome profiling of the Chlamydomonas psb28 mutant reveals TEF5 as an early PSII assembly factor
Source: Plant Cell. 2025 Mar 17;37(6):koaf055. doi: 10.1093/plcell/koaf055 (PMC12164590; doi:10.1093/plcell/koaf055)
Supplement: koaf055_Supplementary_Data [file koaf055_supplementary_data.zip › psb28_interactive.zip!/plots/intensity/qualityPlots/peptides/ASA6_Cre02.g079800.t1.2.html]

### Cre02.g079800.t1.2

Trivial name: ASA6  
SeqCoverage [median]: 59.30  
Mass: 16.05 kDa  
  
 

| Sequence | MissedCleavage | Mass [Da] | wtIntensity | muIntensity | Score |
| --- | --- | --- | --- | --- | --- |
| GMAPATAAPFNGLR | 0 | 1372.69 | 7908.26 | 23689.34 | 179.10 |
| LDTQAR | 0 | 702.37 | 31168.47 | 36575.01 | 137.44 |
| LKPFYPEPAAAAGWSLQAR | 1 | 2072.08 | 5267.32 | 18248.71 | 102.39 |
| SAVALKP | 1 | 684.42 | 0.00 | 2134.13 | 60.36 |
| SVFVALR | 0 | 790.47 | 37015.20 | 81502.59 | 153.61 |
| SVSETSGANLLASLK | 0 | 1475.78 | 45882.26 | 84305.40 | 268.95 |
| SVSETSGANLLASLKAEQAK | 1 | 2003.05 | 33879.92 | 105453.91 | 145.52 |
| TSAASAEESSVANLVK | 0 | 1562.78 | 3054.61 | 7693.47 | 229.29 |
|  |  |  | 164176.0 | 359602.6 |  |
